# Weaker beta desynchronization indicates impaired emotion recognition in schizophrenia

**DOI:** 10.1038/s41537-025-00591-4

**Published:** 2025-03-07

**Authors:** Gábor Csukly, Hajnalka Molnár, Csilla Marosi, Zsuzsanna Fodor, Kinga Farkas

**Affiliations:** Semmelweis University, Department of Psychiatry and Psychotherapy, Budapest, Hungary

**Keywords:** Schizophrenia, Biomarkers

## Abstract

In schizophrenia, deficits in social cognition, such as facial emotion identification, have a significant impact on patient’s daily functioning and quality of life. We analyzed the beta event-related desynchronization (ERD) associated with emotional facial displays to understand better both phase-locked (i.e., neural activity and corresponding EEG response have a fixed latency after the stimulus onset) and non-phase-locked, induced (i.e. the latency of the response is not fixed) electrophysiological correlates of emotion recognition. 128 channels of EEG data from 37 patients with schizophrenia and 40 healthy controls were analyzed. Study groups were matched by sex age, and education. Participants had to identify facial displays of happiness, sadness, and neutral faces from the ‘Karolinska Directed Emotional Faces (*KDEF*)’ database. The time window of 300–700 ms was chosen to analyze spectral perturbation in the beta range associated with the presented emotional faces. Beta desynchronization was observed in both groups. We observed weaker beta ERD in patients. Weaker beta desynchronization correlated with poorer emotion recognition performance in the same time window in the patient group with a maximum correlation at the frontocentral region. Our main finding is that impaired emotion processing in patients with schizophrenia manifested as weaker beta desynchronization when perceiving faces reflecting sad and happy emotions or neutral facial expressions. Furthermore, less prominent beta desynchronization was associated with poorer emotion recognition performance in patients.

## Introduction

Schizophrenia is a disorder characterized by positive, negative, and cognitive symptoms—that affects about 1% of the population worldwide. Emotional recognition deficit is also a well-known symptom of schizophrenia, which is present at the onset of the mental disorder^1^ and can highly affect the patients’ daily functioning^2^. In daily life, the accurate perception and interpretation of emotions is a crucial process in non-verbal communication, social interactions, and interpersonal relationships. Social cognition allows us to decode the emotions, intentions, and mental states of others in our social relationships to understand social interactions^3^. Impairment in social cognition significantly impacts a patient’s quality of daily life and functioning^3–5^. Emotion processing is a fundamental component of social cognition impaired in patients with schizophrenia. Facial emotion recognition impairment is present in patients, their unaffected relatives^6^, and persons vulnerable to psychosis^7^. Though it is a widely investigated topic, the exact electrophysiological and neurobiological background of this impairment is still unclear. Therefore, we analyzed the electrophysiological, oscillatory background of facial emotion processing in schizophrenia patients and healthy control subjects.

In order to understand the electrophysiological background of the neural cascade process required for the recognition of facial emotions, previous studies have focused on event-related potentials (ERPs)^8–11^. Luo et al. analyzed the processing of facial emotions using the ERP technique and then divided the process into three main stages: the first stage involves rapid and automatic processing of stimuli. At this point, the brain already separates negative stimuli from other stimuli. The potential emotional valence is determined in the second stage, while in the third and final stage, facial emotions are isolated and accurately identified^9^.

Given the temporal resolution of the ERP technique, the measured changes are time-locked (i.e., neural activity and corresponding EEG signal change follow the stimulus at exactly the same time: the response has a fixed latency after stimulus onset) and closely related to the presented stimulus. Consequently, the brain’s electrophysiological responses to visual or auditory events are not entirely reflected in the ERP. This would only hold true if (1) evoked responses were consistent ( ~ time-locked) across trials, (2) entirely independent of the ongoing (background) EEG, and (3) the ongoing EEG itself was unaffected by experimental events. However, studies^12,13^ have demonstrated that ERPs are neither consistently stable nor entirely independent of the ongoing EEG. Consequently, the brain’s response to visual or auditory events cannot be fully represented by the ERPs^14,15^. In contrast, with the event-related spectral perturbation (ERSP) technique, we can gain insight into the triggered and the non-phase-locked, induced neural activity (i.e. neural activity and corresponding EEG signal change does not follow the presented stimulus at exactly the same time: response latency after stimulus onset is not fixed). ERSP measures the relative changes of a given spectral power relative to baseline, thus allowing the study of the time course of the EEG signal in specific frequency bands. ERSP estimates the change of EEG power over frequency and time^14,15^ giving a more comprehensive image of the EEG activity induced by the experimental event. A further advantage of ERSP is that measuring a relative shift in baseline EEG activity is more robust towards phenomena such as low voltage. A positive change in ERSP is referred to as event-related synchronization (ERS, while a negative change in ERSP is referred to as event-related desynchronization (ERD) in the literature.

Previous research has shown that the beta frequency range (12–30 Hz) is associated with perception, attention, and cognition. Oscillatory activity in the beta range also plays an essential role in the perception of facial and emotional images^16–21^. Several research groups have investigated beta oscillation using the International Affective Picture System (IAPS) image collection and presenting photographs of faces, with reduced beta responses found for negative or unpleasant stimuli compared to neutral or positive stimuli^18,22,23^. Previous studies also showed that facial emotion processing is reflected in beta (12–30 Hz) and in low beta (13–18 Hz) desynchronization^17,18,24^. Huebl and colleagues described a significantly stronger beta ERD (i.e., a more pronounced negative change) during the presentation of emotionally salient pictures compared to neutral ones^25^. Schubring and colleagues examined the effect of the potency of the presented emotions. Their results showed that high-arousing pictures elicited a stronger ERD response in the beta range^26^. These findings suggest a connection between the decrease of beta power (i.e., stronger ERD) and emotion processing.

Our group has previously analyzed impaired emotion recognition using ERSP. In schizophrenia, we investigated the oscillatory processing background of fear-expressing and emotion-neutral faces; we found reduced theta synchronization in the schizophrenic group compared to the control group in the 140–200 ms time window. We observed stronger theta synchronization for faces expressing fear than for neutral faces. In both groups, we found a correlation between synchronization and performance on the emotion recognition task^27^. We then examined emotion processing using IAPS images; we found reduced low-beta desynchronization in schizophrenia compared to the healthy control group. Beta ERD correlated with negative symptom severity and psychosocial functioning^22^. In our latest publication, we focused on the oscillatory background of early visual perception (100–200 ms) and the decoding phase of faces (140–200 ms), detecting reduced theta synchronization in the emotion recognition paradigm in schizophrenia compared to the control group^28^.

In the present study, we used the ERSP technique to investigate the third stage of facial emotion processing, the beta oscillation associated with the differentiation between emotions and their processing. Based on the emotion processing dysfunction observed in schizophrenia, we expected reduced beta-desynchronization in patients compared to healthy control subjects. Furthermore, based on our previous results, we hypothesized that reduced beta-ERD would correlate with negative symptom severity in schizophrenia.

## Methods

### Participants

The study included 37 patients with schizophrenia, including eight patients with schizoaffective disorder, and 40 age-, sex- and education-matched controls. Most participants were right-handed, except for three left-handed and one ambidextrous patient and three left-handed and two ambidextrous control subjects. All participants had normal or corrected to normal vision with glasses/contact lenses.

The patients were selected from the patients of the Department of Psychiatry and Psychotherapy of Semmelweis University. The diagnosis was based on the DSM-V^29^ diagnostic criteria system. The severity of psychiatric symptoms was assessed using the PANSS—Positive and Negative Syndrome Scale^30^, administered to patients by psychiatrists trained and skilled in the instrument. During the study, the patients did not stop taking their regular medication, and all patients received antipsychotic treatment. The chlorpromazine equivalent^31^ mean dose was 517.9 mg/day (SD = 338 mg).

Exclusion criteria for the study included severe central nervous system, neurological disease (e.g., stroke, tumor, epilepsy, Parkinson’s disease, dementia), mental retardation, drug or alcohol use disorder, and abuse of these stimulants in the three months prior to the study. Furthermore, a history of head injury associated with loss of consciousness for more than 1 hour was an exclusion factor. Any psychiatric illness in the medical history was an exclusion factor for healthy controls. In addition, if the control subjects had completed the Derogatis SCL-90R (Symptom Checklist - 90 R) questionnaire^32^ and exceeded the threshold previously set for the Hungarian sample ( > 114), they were excluded from the study.

The study was approved by the Regional, Institutional Scientific, and Research Ethics Committee of Semmelweis University in Budapest. Written informed consent was obtained from participants prior to the studies. The research was conducted in full compliance with the Declaration of Helsinki.

Demographic data of the two groups and the main clinical characteristics of patients with schizophrenia are shown in Table 1.

**Table 1:** Demographics and clinical characteristics.

	Patients with schizophrenia	Healthy controls	statistics	*p*-value
Sex (male/female)	22/15	25/15	Chi2 = 0.07	ns.
Age	33.8 (10.6)	32.8 (9.6)	*t* = −0.27	ns.
Education^a^	3/24/10	0/28/12	Fisher exact test	ns.
Time since onset of illness (years)	8.6 (8.7)	-		
Inpatient/outpatient/day hospital	4/16/17	-		
CPZ equivalent dose	517.9 (344)	-		
PANSS total score	62.9 (16.6)	-		
PANSS positive score	15.4 (4.6)	-		
PANSS negative score	16.2 (5.8)	-		
PANSS overall score	31.3 (8.2)	-		

*CPZ* Chlorpromazine, *PANSS* Positive and Negative Syndrome Scale.

^a^1 = < 8 grades (primary); 2 = secondary school (intermediate); 3 = college/university (advanced).

### Procedures

During the EEG (Electroencephalography) recording, participants were seated in a dimly lit, quiet room. The applied visual stimuli were presented on a computer monitor using Presentation 13.0 software (Neurobehavioral Systems, Inc.; Albany, CA). Participants were positioned at a distance of half a meter from the monitor.

**Fig. 1:**
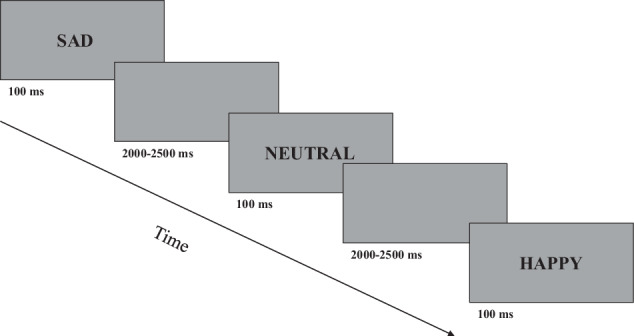
Schematic arrangement of the emotion recognition paradigm. Participants had to separate faces expressing emotionally neutral, happy, and sad emotions by pressing a button. Stimuli were displayed on the screen for 100 ms. The time between the presentation of the two stimuli varied between 2000ms and 2500 ms in a randomized manner.

In the emotion recognition paradigm, faces expressing happy and sad emotions and neutral faces were presented to the participants in 5 blocks. The images of eight female and eight male faces were presented using the Karolinska Directed Emotional Faces (KDEF)—image collection^33^. Each face was viewed horizontally and vertically at a 6° viewing angle from the screen. The stimuli were displayed on a grey background and were visible for up to 100 ms. For asynchronous stimulus alternation, the time between stimuli was randomized between 2000 and 2500 ms. During the task, participants were asked to indicate the emotion they saw on displayed faces by pressing a button in response to the stimuli. The participants could lock in their answers during the time interval between the presented pictures (2000–2500ms). A total of 16 happy, 16 neutral, and 16 sad faces appeared per block (altogether 240 facial displays). A schematic of the test design is shown in Fig. 1.

### EEG recording and pre-processing

Recordings were acquired from DC using a BioSemi ActiveTwo amplifier with 100 Hz low-pass filtering^34^. A 128-channel cap with high electrode density was used to derive EEG from the entire scalp area. We recorded electrooculograms with two electrodes (located below the left canthus and above the right canthus) to record horizontal eye movements. Sampling was performed at a frequency of 1024 Hz.

Built-in and self-developed Matlab functions for off-line data analysis (MathWorks, Natick, MA) and the free-to-use toolkit of EEGLAB software^35^ were used. Pre-processing was performed before statistical analysis of the raw electrophysiological data that involved referencing the recordings to a common average potential and filtering off-line by a band-pass filter between 0.1 and 45 Hz.

Each registry was also reviewed manually to exclude bad channels. Epochs containing motion and other artifacts or technically inappropriate drains were individually selected and interpolated using data from the surrounding electrodes. Motor (muscle and eye movement) artifacts were removed using ADJUST, an automated artifact detection software based on Independent Component Analysis (ICA)^36^. In addition, we also removed artifacts exceeding ±100 μV on any EEG or EOG leads.

Altogether 80 happy, 80 neutral, and 80 sad faces were presented in both study groups. After artifact removal, an average of 71.8 (SD = 11.4) sad, 71.8 (SD = 12.1) neutral, and 71.8 (SD = 12.0) happy trials have left in the control group. While for patients, 65.7 (SD = 15.9) sad, 66.5 (SD = 13.9) neutral, and 67.0 (SD = 13.6) happy trials were included in the statistical analysis.

**Fig. 2:**
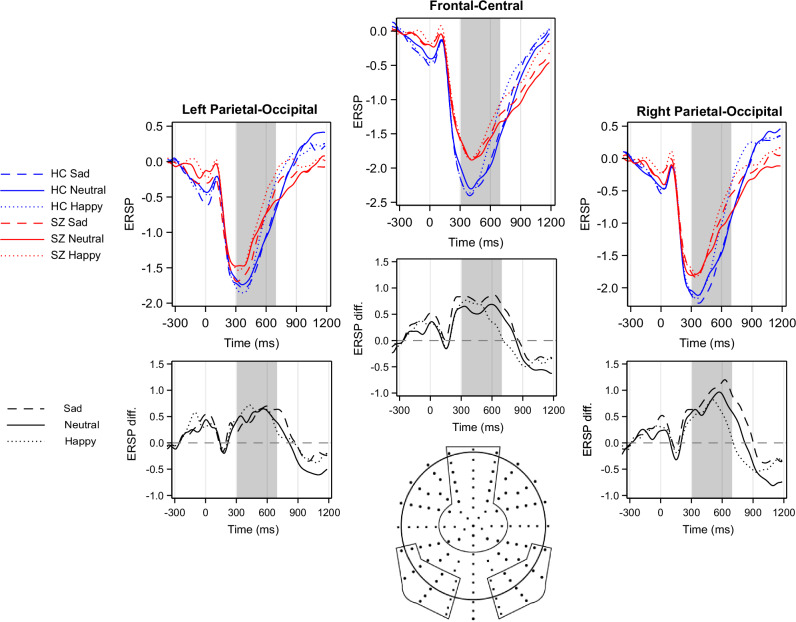
Event-related desynchronization (ERD) in the beta frequency range for the three emotional conditions and the two study groups. The differences between study groups in beta ERD are presented below the ERD plots: patients relative to controls: prominent positive deflections mean more negative values (i.e. stronger ERD) in controls. The regions of interest (ROIs) selected for analysis are indicated by circled areas on the scalp map. HC Healthy Control, SZ Patients with Schizophrenia, diff. difference.

Electrodes were grouped into three regions of interest (ROI): a fronto-central, a right temporo-parietal-occipital, and a left temporo-parietal-occipital location (Fig. 2). The regions were chosen based on previous studies applying similar experimental designs^22,37,38^, and are in line with a priori knowledge that temporo-parieto-occipital and frontal regions are vital in facial expression recognition and emotion processing. The ERSPs from all channels were averaged over a given ROI to reduce noise further.

### Statistical analysis

To investigate the stimulus-related beta (13–30 Hz) activity change, we used the Event-Related Spectral Perturbation (ERSP) method, which provides a two-dimensional representation of the average change in spectral power relative to baseline power^39^. The baseline power for ERSP calculation was determined from the EEG curve immediately preceding the stimulus presentation, which was divided into short overlapping windows and averaged over their varying amplitude spectra. Each spectrally transformed section was normalized by its mean spectrum. The normalized ERSP sections of the trials were averaged and plotted as the logarithm of the relative spectral amplitude on a time-frequency plane^39^.

ERSP analysis was performed from 600 ms pre-stimulus to 1400 ms post-stimulus. The time window was 400 ms long and was applied 200 times. A baseline correction was performed by averaging the 600 ms to 200 ms pre-stimulus intervals. To calculate the average ERSP value, the ROIs were averaged together to reduce noise further.

According to previous research investigating beta oscillations in emotion processing paradigms^22^, emotion processing starts after face recognition and is delayed in time. Therefore, a time window of 300–700 ms was chosen for further data analysis to capture oscillatory activity connected to emotion processing.

The differential effects of the study group (healthy control (HC vs. schizophrenia (SZ) × ROI (frontocentral, right temporoparietal-occipital, left temporoparietal-occipital)) × stimulus type (sad vs. neutral vs. happy) on ERSP were investigated using a repeated measure mixed model analysis. The mixed model included both main effect and two-way and three-way interactions. To examine the interactions, pairwise post-hoc analyses were performed. Given that comparisons between groups were made across three regions, the Bonferroni correction for multiple comparisons was used in the post-hoc analysis. Since study groups differed in reaction times, and movement may affect beta ERD, we included reaction times as a covariate in the mixed model analyses.

The correlation of emotion recognition task performance with ERSP scores was examined using Spearman correlation, as the behavioral scores deviated from the normal distribution. The relationship between PANSS scores and ERSP was also examined using the Spearman correlation.

## Results

### Behavioral data

During the test, patients responded to presented faces by pressing response buttons. The distribution of the behavioral results deviated from the normal distribution, so a non-parametric Mann-Whitney U-test was used. There were significant differences between the test groups regarding accuracy and reaction time. Behavioral data for the emotion recognition task are shown in Table 2. Marosi et al.^28^ reported the same behavioral results previously.

**Table 2:** Results of the emotion recognition task.

	Patients with schizophrenia (*n* = 37)	Healthy controls (*n* = 40)	Statistic	*p*-value
Total score	80.0% (10.9)	89.1% (3.7)	*U* = 305	<0.0001
Sad	76.7% (12.3)	85.7% (6.3)	*U* = 421	<0.001
Neutral	75.2% (19.0)	88.2% (5.9)	*U* = 394	<0.0004
Happy	88.4% (10.3)	93.9% (3.1)	*U* = 506	<0.02
Reaction time	784.8 ms (128.1)	714.2 ms (77.3)	*t* = 2.95	0.004

### Beta desynchronization in the 300–700 ms time window

A decrease in ERSP, i.e., Event-Related Desynchronization (ERD), was observed in both study groups (difference from zero in beta ERD: control group: *t* = −11.2, *p* < 0.0001; patient group: *t* = −7.7, *p* < 0.0001) in the 300–700 ms time window (Fig. 2).

A significant between-group difference was detected (F(1;74) = 4.8, *p* = 0.03), indicating that beta desynchronization was weaker in patients compared to controls. The main effect of stimulus condition was also found to be significant (F(2,74) = 12.6, *p* < 0.0001). The post-hoc tests demonstrated a significant difference between neutral and happy conditions (*t* = −5.0, *p* < 0.0001) and between sad and happy conditions (*t* = −3.1, *p* = 0.003). The difference between sad and neutral conditions showed a statistical trend (*t* = −1.8, *p* = 0.08). Region of interest was also found to be significant F(2,74) = 24.5, *p* < 0.0001 (Right Temporo-Parietal > Fronto-Central > Left Temporo-Parietal region). The effect of reaction time was not significant (F(1;74) = 0.7, *p* = 0.4). None of the interactions were found to be significant.

#### Scalograms depicting ERSPs in all analyzed regions and for all stimuli are presented as Supplementary Fig. S1

##### Correlational analyses

**Fig. 3:**
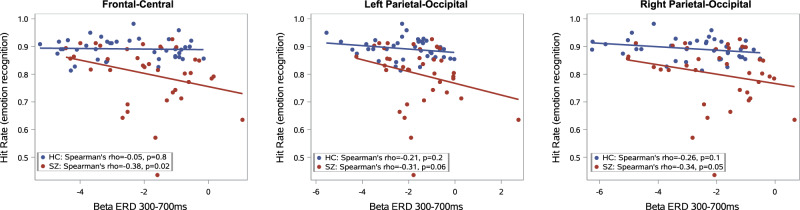
Correlation between beta ERSP and total emotion recognition task score during the second (700–900 ms) time window. (‘r’ indicates Spearman rho correlation coefficient).

Emotion recognition task performance (averaged over all facial displays) significantly correlated with beta desynchronization (averaged over all types of stimuli) in the fronto-central (**All subjects**: Spearman’s *r* = −0.28, *p* = 0.01; **SZ**: *r* = −0.37, *p* = 0.02; **HC**: *r* = −0.05, *p* = 0.8), in the left temporo-parietal-occipital region (**All subjects**: *r* = −0.28, *p* = 0.01; **SZ**: *r* = −0.31, *p* = 0.06; **HC**: *r* = −0.21, *p* = 0.2), and in right temporo-parietal-occipital (**All subjects**: *r* = −0.34, *p* = 0.003; **SZ**: *r* = −0.32, *p* = 0.05; **HC**: *r* = −0.26, *p* = 0.1) regions (Fig 3). Our results indicate that weaker beta desynchronization is associated with poorer emotion recognition performance. Correlations of ERD with hit rates for each emotion separately are presented in the Supplementary Material Tables S1-S6. No significant correlation was found between beta ERD and reaction times in any region (*p* > 0.1 in all ROIs).

Antipsychotic dose in terms of Chlorpromazine equivalents^32^ and PANSS showed no correlation with beta desynchronization in any region (*p* > 0.1).

## Discussion

Patients performed significantly slower and worse on emotion recognition than controls on all three stimulus types. Furthermore, these behavioral findings correlated with beta ERD. Our behavioral results are consistent with previous emotion recognition tasks that have described impaired emotion recognition in schizophrenia^9,39^. Recognition of emotionally neutral faces and faces expressing sadness was more difficult for patients than identifying faces expressing happiness; our results are consistent with those of previous studies that have described a more severe recognition impairment for negative emotions^28,40^.

From previous functional MRI studies, it is known that social cognition, including face processing, is primarily associated with temporo-parietal regions^34,35^, whereas emotion recognition and processing are associated with basal ganglia, parietal cortex, medial temporal areas, cingulate, amygdala, and prefrontal cortex^36^. Previous neuroimaging studies described a decreased activation of these brain regions, often called “the social brain” in patients with schizophrenia during emotion recognition tasks^41^. MRI can provide a more accurate picture of which neurobiological processes are associated with which neural structures, however the temporal resolution of MRI is low. Electrophysiological studies can provide insights into the temporal dynamics of these processes. The electrophysiological background of the neural processes involved in emotion recognition has been analyzed using event-related potentials (ERPs), for which significant differences were found between healthy controls and patients with schizophrenia in several ERP components (P100, N170, N250)^6,8,9,22,37^. Subsequently, there has been increasing interest in the more precise mapping of central nervous oscillations associated with higher cognitive processes, including beta and theta frequency bands^17–20,22,27,42^.

In the present study, we analyzed the evoked and induced neural activity by calculating the event-related spectral perturbation (ERSP) to get a more accurate picture of the electrophysiological activity associated with emotion processing. The coordinated activity of neural oscillations, which are related to the inhibition and excitation of neural networks, plays a crucial role in physiological neural function^43^. In recent years, the analysis of oscillations has received increasing attention, not only in healthy but also in patient populations, since ERSP analyses allow, in particular, the isolated analysis of specific frequency bands, including beta and gamma, which is impossible in traditional ERP analyses. They also allow the study of not only evoked but also stimulus-induced brain activity^14^. Based on these characteristics, they may even be potential biomarkers for individual patients’ detection, isolation, and follow-up.

We observed a desynchronization (ERD) of the beta frequency band (13–30 Hz) for faces expressing neutral, happy, and sad emotions. In patients, decreased beta desynchronization detected in the first time window (300–700 ms) was associated with poorer emotion recognition performance. This finding aligns with the results of Merkl et al., who found stronger beta desynchronization in patients with a major affective disorder and higher empathic skills^44^. Our results support that beta oscillation reflects, among other things, the processing of emotions^14,43^. However, we only found a trend-level correlation (*p* = 0.01) between beta ERD and behavioral performance in the control group, which may be due to the smaller variance in performance in the healthy group.

MEG studies tried to link the neurobiological basis of beta suppression in facial expression processing and found that the possible generators of beta ERD are frontal areas, the amygdala, and the fusiform gyrus. Senkowski et al.^45^ and Jiang et al.^46^ found that viewing emotional facial stimuli led to beta suppression (ERD) in the somatosensory cortex, while Qing et al. found that after viewing sad facial stimuli the connectivity between the anterior cingulate cortex and the amygdala showed a decrease in beta power^47^. Schneder et al. also linked beta suppression during facial expression processing to the activity of the amygdala and the fusiform gyrus^48^. Another possible mechanism for beta ERD in processing human facial expressions is proposed by Cooper et al.^49^, who connected the desynchronization in beta oscillations to mirror neuron (Brodmann area 44) activity during facial expression processing. However, further studies applying MEG, or concurrent EEG and fMRI imaging, are needed to investigate the exact neurobiological underpinning of beta ERD during facial expression processing.

There is a growing literature regarding the treatment options for schizophrenia patients with emotion recognition impairment. According to Tsotsi and colleagues, targeted training can significantly improve patients’ emotion recognition skills^50^. Intranasal oxytocin admission was also found to be useful in clinical trials^51,52^. Transcranial direct current stimulation (tDCS) and repetitive transcranial magnetic stimulation (rTMS) showed significant effect on facial emotion recognition in previous studies in schizophrenia, although the results are not consistent, further investigation is needed in this field^53^. Considering these results, beta desynchronization could also be a helpful biomarker for treatment development.

Our present study has some limitations; all patients were on antipsychotic medication during the study, while controls were free of psychotropic medication. However, we did not find a significant correlation between equivalent doses of chlorpromazine and the rate of beta-desynchronization in any of the study regions. The EEG experiment was long and complex; therefore, only well-cooperating patients with symptom severity scores in the low/medium range (mean PANSS score was 62.9) were included. Our study only examined three stimulus types—happy, sad, and neutral - and did not address the other basic emotions.

## Conclusions

Overall, the results of our study confirm that beta-desynchronization is related to emotion processing and that weaker beta-desynchronization in patients with schizophrenia correlates with poorer emotion recognition and more severe emotional blunting.

## Supplementary information

Supplementary Material

Competing interest form

## Data Availability

The datasets that are used and/or analyzed during the current study are available from the corresponding author on reasonable request.
