# Supplementary material for: Weaker beta desynchronization indicates impaired emotion recognition in schizophrenia
Source: Schizophrenia (Heidelb). 2025 Mar 7;11(1):39. doi: 10.1038/s41537-025-00591-4 (PMC11889095; doi:10.1038/s41537-025-00591-4)

Supplementary material

Weaker beta desynchronization indicates impaired emotion recognition in schizophrenia

S1. <u>Correlation analysis between hit rates and beta desynchronization (ERD)</u>

Spearman correlation analyses between hit rates for all presented emotions and the corresponding beta ERD in the study groups. Significant ($p<0.05$) and marginally significant ($p<0.1$) correlations were marked with **bold**.

<u>S1.1. *Correlation between hit rates and beta ERD in the* control group</u>

Spearman's correlation coefficient (p value)	***Beta ERD for happy pictures***	***Beta ERD for neutral pictures***	***Beta ERD for sad pictures***
Hit rate for happy pictures	0.056 (0.732)	-0.032 (0.845)	0.005 (0.978)
Hit rate for neutral pictures	-0.175 (0.281)	-0.212 (0.19)	-0.195 (0.228)
Hit rate for sad pictures	0.102 (0.533)	0.072 (0.657)	-0.03 (0.854)

Table S1: Correlation between hit rates and beta ERD in the 300-700 ms time window in the frontocentral region in the control group. (p- values in brackets)

Spearman's correlation coefficient (p value)	***Beta ERD for happy pictures***	***Beta ERD for neutral pictures***	***Beta ERD for sad pictures***
Hit rate for happy pictures	-0.238 (0.14)	-0.21 (0.193)	**-0.293 (0.066)**
Hit rate for neutral pictures	**-0.38 (0.016)**	**-0.306 (0.055)**	**-0.309 (0.053)**
Hit rate for sad pictures	0.004 (0.98)	0.017 (0.917)	0.033 (0.842)

Table S2: Correlation between hit rates and beta ERD in the left temporoparietal region in the 300-700 ms time window in the control group (p- values in brackets)

Spearman's correlation coefficient (p value)	***Beta ERD for happy pictures***	***Beta ERD for neutral pictures***	***Beta ERD for sad pictures***
Hit rate for happy pictures	-0.261 (0.104)	**-0.292 (0.068)**	0.234 (0.147)
Hit rate for neutral pictures	**-0.336 (0.034)**	**-0.354 (0.025)**	**-0.32 (0.045)**
Hit rate for sad pictures	-0.052 (0.752)	-0.06 (0.714)	-0.098 (0.547)

Table S3: Correlation between hit rates and beta ERD in the right temporoparietal region in the 300-700 ms time window in the control group (p- values in brackets)

S1.2. *Correlation between hit rates and beta ERD in the patient group*

Spearman's correlation coefficient (p value)	***Beta ERD for happy pictures***	***Beta ERD for neutral pictures***	***Beta ERD for sad pictures***
Hit rate for happy pictures	-0.245 (0.144)	**-0.293 (0.078)**	**-0.308 (0.063)**
Hit rate for neutral pictures	-0.068 (0.689)	-0.267 (0.11)	-0.153 (0.365)
Hit rate for sad pictures	-0.195 (0.248)	-0.189 (0.261)	-0.105 (0.537)

Table S4: Correlation between hit rates and beta ERD in the 300-700 ms time window in the frontocentral region in the patient group. (p- values in brackets)

Spearman's correlation coefficient (p value)	***Beta ERD for happy pictures***	***Beta ERD for neutral pictures***	***Beta ERD for sad pictures***
Hit rate for happy pictures	-0.181 (0.285)	**-0.316 (0.056)**	-0.267 (0.11)
Hit rate for neutral pictures	-0.135 (0.425)	**-0.295 (0.077)**	-0.202 (0.231)
Hit rate for sad pictures	0.039 (0.82)	-0.049 (0.773)	0.012 (0.942)

Table S5: Correlation between hit rates and beta ERD in the left temporoparietal region in the 300-700 ms time window in the patient group (p- values in brackets)

Spearman's correlation coefficient (p value)	***Beta ERD for happy pictures***	***Beta ERD for neutral pictures***	***Beta ERD for sad pictures***
Hit rate for happy pictures	-0.153 (0.365)	**-0.306 (0.065)**	**-0.282 (0.091)**
Hit rate for neutral pictures	-0.099 (0.559)	-0.255 (0.127)	-0.221 (0.188)
Hit rate for sad pictures	-0.053 (0.754)	-0.109 (0.519)	0.001 (0.993)

Table S6: Correlation analysis results in the right temporoparietal region in the 300-700 ms time window in the patient group (p- values in brackets)

Figure S1: Scalograms of the time frequency analysis (HC = Healthy Controls, SZ = Patients with Schizophrenia)

Scalograms were obtained by the wavelet transformation of the EEG signal. Scalograms represent the change of the EEG power in the certain frequency ranges over time. The horizontal axis indicates the time (ms), the vertical axis the frequency (Hz), and the color intensity indicates spectral power change (relative to baseline) of the signal at the given time and frequency points.

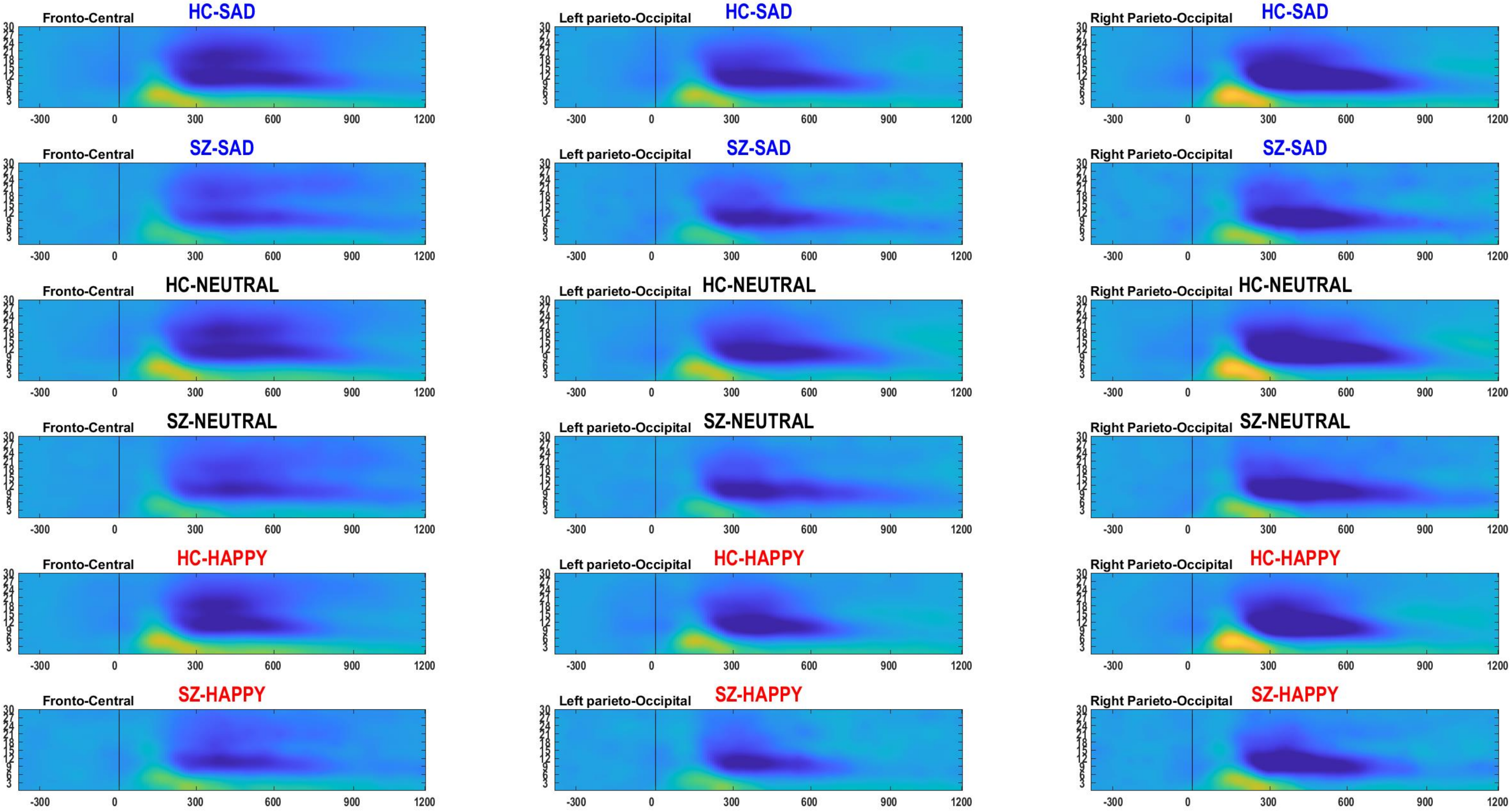

Supplement: Supplementary file 1 — Supplementary Material [file 41537_2025_591_MOESM1_ESM.pdf]
